# Time trend analysis of breast cancer in Iran and its six topographical regions: a population-based study

**DOI:** 10.25122/jml-2018-0087

**Published:** 2019

**Authors:** Sattar Bab, Edris Abdifard, Shahin Elyasianfar, Payam Mohammadi, Mohammad Heidari

**Affiliations:** 1. Students Research Committee, Kermanshah University of Medical Sciences, Kermanshah, Iran; 2. Iran Nursing and Midwifery School, Iran University of Medical Sciences, Tehran, Iran; 3. Clinical Research Development Unit of Imam Khomeini Hospital, Urmia University of Medical Sciences, Urmia, Iran

**Keywords:** breast cancer, incidence rate, Iran, trend

## Abstract

Breast cancer is the most common cancer among women, and in 2002 it was expected that 636,000 new cases would occur in developed countries and 514,000 in developing countries. Although the incidence rate of this cancer in Asian countries is lower than in Western countries, whereas the incidence trend increasing rapidly in Asia. Using the data from the Cancer Registry System, this study was carried out to investigate the incidence trend during 2000–2005 in Iran and its six geographical areas. The incidence rates were standardized according to age-sex groups by Excel directly and confidence intervals is calculated for the point estimations by Stata11. The trends were analyzed separately based on gender, age groups for different provinces by Poisson regression in Stata11. The age standardized incidence rate in 2000 was 0.1 and 3.4 per 100,000 in males and females, respectively, reaching 0.5 and 16.7 per 100,000 in 2005. The incidence trends in all geographical areas of the country were increased despite the difference in the slopes. The sex ratio of male to female was 31.6 and the mean age of the patients was 49.4 (±12.6) years. The incidence of breast cancer in Iran was lower than the European and Asian countries but the trend is in rising. Although this increase is due to the increase in the prevalence of risk factors among Iranian population. Improvement in the coverage of the cancer registry system as well as the screening programs are important factors for these changes.

## Introduction

The rapid growth of cancers are causing a crisis in public health and the health system around the world, and it is expected that 25 million new cases of cancer and 17 million deaths from cancers will occur every year. The highest increase in cancers cases will be seen in the Third World countries with low or moderate income [1].

Breast cancer is the most common cancer among women, and in 2012 it was anticipated that 793,000 new cases would occur in developed countries and 882,000 would occur in developing countries [2]. In 2018, 266,120 new cases of breast cancer were predicted for women in the United States and 2550 cancer cases were predicted for men, and breast cancer can be considered the most common cancer among women [3]. It is a disease of women and rarely occurs in men; i.e. only 1% of breast cancer cases occur among men [4].

In addition to female sex, age is the most important risk factor for breast cancer, and other risk factors include high weight or obesity, estrogen or progesterone therapy, lack of physical activity and alcohol consumption [3]. In women, each year of delay in the menarche may reduce the incidence of breast cancer by 15%, and each year of delay in menopause may increase it by 3% [5]. On the contrary, breastfeeding is a protective factor against breast cancer, and studies have shown that for each year of breastfeeding, the risk of developing this cancer would reduce by 3% to 4% [6]. One of the unchangeable risk factor for breast cancer is the family history, because the chance of developing breast cancer in the women with family history of the disease (sister, mother and daughter) was twice as much as in those without this family history [3].

The highest prevalence rates (89–96 cases per 100,000 people) have been seen in Northern and Western Europe, North America, and Australia/New Zealand [2], but the incidence trend has grown rapidly in developing countries in recent decades, while this trend was slower in advanced countries [1]. Breast cancer risk factors in Iran are not different from those in other parts of the world, and include obesity, menopause and having family history [7]. Moreover, the 5-year survival rate is low in Iranian patients due to the diagnosis in advanced stages of disease [8, 9]. Breast cancer is the most common cancer among Iranian women, but its incidence rate is lower than the global average and in countries such as the United States and Western Europe [10], and the rate of Iranian women immigrants to the Western countries has increased four-fold [11]. A study in 5 educational hospitals in Tehran showed that breast cancer cases in Iran were younger than in Western countries, but on the contrary, they were mainly suffering from advanced stages of the disease. That is to say, the mean age of breast cancer was 47.1 with a standard deviation of 12.3 years, and 70% of the cases were in advanced stage of the disease [12].

The trend analysis can provide researchers with clues to better understand the causes for this cancer. Fortunately, many studies have been conducted on breast cancer in Iran, and various aspects of the epidemiology of this cancer have been found out, and it is also recommended that future studies in Iran address the clinical aspects of the cancer [13]. In Semnan, too, some studies were carried out on cancer during 1998–2002 and it was found out that breast cancer was the most common among women [14]. Besides, a population-based study in 5 provinces of Iran during 2003–2006 showed that breast cancer was the most common cancer in these provinces with the total incidence of 24% [15].

However, in comparison with previous studies, it can be said that breast cancer has had an increasing trend in Iran. In a study on cancers in Tehran in 1970, breast cancer accounted only for 5% of all cancers and was the fifth most common cancer in Iran [16]. A review of the population-based data from the cancer registry in Tehran during 1998–2001 with the aim of determining the breast cancer burden indicated that there were 17 per 100,000 women with breast cancer whose 5-year survival rate was 75%, 31% of whom were under the age 40 years, and the The disability-adjusted life years (DALYs) for this cancer was 42–52 years. In a case-control study in Iran, it was observed that marital status (never married OR=4.2) and family history (OR=2.9) were the only incremental variables of breast cancer in individuals [17].

Various studies have identified the pattern of breast cancer incidence in Iran. A study on the pattern of breast cancer incidence in Tehran during 1986–2000 showed that, with a mean age of 47.9 and a standard deviation of 12.4 years, the rate of diagnosis in the early stages of the disease increased over time and the cases of advanced disease also decreased. The researchers believed that it was due to an increase in health awareness in Iran and the interventions of the health system [18]. The health system interventions have been effective in controlling breast cancer, and the 5-year survival rate of the disease in the United States has risen from 68% in 1960 to 89% in 2008 [3]. The most important program for controlling breast cancer is the screening program for this cancer, which is currently underway in Iran. But evidence suggests that getting involved in routine breast cancer screening programs is not regularly done in Iran, and people delay the visits eventually they die [19]. A study showed that 25% of women delayed the next visit for more than 3 months [20], and despite having proper knowledge and attitudes few health personnel used breast cancer screening programs [21]. In this regard, another study among on female health staff in Tehran showed that although 63% of them were familiar with breast cancer self-examination, only 6% did it on a monthly basis [22].

In the screening program in Iran, the highest participation rate is that of the women aged 35–44 years and from a middle socioeconomic class, and the lowest participation rate is seen among those over the age of 60 years and from a low socioeconomic class. Furthermore, the rate of screening in the self-examination method is similar to that done by the health personnel [23]. Fortunately, there are not many problems in terms of the mammography in Iranian women, and most of them do not consider it to be against the Islamic beliefs [24]. However, the knowledge of ordinary women about the symptoms and screening programs of breast cancer is very low and there is still a dire need for educating them [25]. Psychosocial factors have been associated with an increase in breast cancer in Iranian women as well [26].

Cancers are the third leading causes of death and one of the research priorities in Iran’s health system. In spite of dispersed and provincial studies on the prevalence of cancers, there has been no comprehensive study using national data on the changes in the incidence trend of breast cancer in Iran, and no documentary evidence of the changes in this trend is available in the country. Since the future control and planning of the health system in order to reduce the incidence of breast cancer requires the information on the epidemiological situation of this cancer and its incidence trend in recent years, the present study was carried out to investigate the age standardized incidence rate of breast cancer during 2000–2005 for the first time, based on the data from the National Cancer Registry System of the country and in 7 different geographical areas of the country, separately. The results of the present study could help the health system policy makers (with the analysis of cancer care system), the effects of health interventions and the future prediction of breast cancer status in Iran.

## Materials and Methods

In this population-based cross-sectional study, the data were obtained from the national cancer registry system of the Disease Management Center affiliated to the Ministry of Health. At the end of each year these data are collected from pathology centers all over the country, after which the Disease Management Center reviews them for correct coding, deficiencies in identity and demographic information, and removal of the repeated cases reported. It is worth noting that cancers have been coded according to the International Classification of Diseases (ICD_O, Second Edition) [27]. In this study, the code C50 was extracted for breast cancer. We divided the country into six climatic regions. Thus, we selected Gilan and Mazandaran provinces as the central and western borders of the Caspian Sea (North West), Golestan province alone as the eastern border of the Caspian Sea (North East), the provinces of Tehran, Qom, Qazvin, Zanjan, East Azerbaijan, West Azerbaijan and Ardebil as the flat area, the provinces of Kermanshah, Kurdistan, Hamedan, Ilam, Chahar Mahal and Bakhtiari, Kohkiloyeh and Boyer Ahmad, and Fars as mountainous area, Kerman, Sistan and Baluchestan, Yazd, Semnan, Khorasan, Birjand and Isfahan provinces were selected as desert area, and ultimately the provinces of Khuzestan, Bushehr and Hormozgan were selected as the Persian Gulf area. However, the residence of a large number of recorded cancer cases was unknown, and they were included in the calculation of the total incidence in the country but not in the calculations of different geographical areas.

To standardize the incidence rates, the results of the national population census in 2006 were used. To do so, the populations of different geographical areas for the years 2000, 2001, 2002, 2003, 2004 and 2005 were estimated by calculating the annual population growth of 1.01% and based on the population of the country in 2006. Also, the WHO standard population was used as a standard population. The sex-age incidence rates and their 95% confidence intervals (CI) for the whole country were calculated directly for each geographical area through the use of the Stata11 software, and presented separately for different age and sex groups. The significance of the incidence trends was tested using the Stata11 software and the Poisson regression model, and the alpha error value of lower than 0.05 was considered significant. The Excel 2007 software was used to draw the diagrams.

## Results

A total of 20,791 cases of breast cancer had been recorded for these 5 years in Iran, which had increased from 1219 cases in 2000 to 6156 in 2005, and the sex ratio of women to men was 31.6. The mean age of the subjects was 49.4 with a standard deviation of 12.6 years. The mean age of the women was 49.1 with a standard deviation of 12.5 years, and that of the men was 57.9 with a standard deviation of 14.7 years. The age standardized incidence rate of breast cancer in Iran had a significant upward trend, with an increase in the total of 1.8 cases per 100,000 people of both sexes in 2000 to 8.6 per 100,000 in 2005. The age standardized incidence rate in women was much higher than in men, so that the incidence rate in women increased from 3.4 per 100,000 individuals in 2000 to 16.7 per 100,000 in 2005, while in men, it increased from 0.1 per 100,000 people in 2000 to 0.5 per 100,000 in 2005 (Table 1).

**Table 1:** Trend of changes in the age-standardized incidence rate (per 100,000 persons) of breast cancer in six geographical areas in Iran during 2000–2005.

	2000	2001	2002	2003	2004	2005	Slope/P Value
**North West**							
Male	0.1 (0.02–0.3)	0.04 (0.008–0.1)	0.8(0.4–1.1)	0.5(0.2–.07)	0.4(0.2–0.7)	0.4(0.1–0.6)	**0.17(0.6)**
Female	4.2(3.4–5.0)	2.1 (1.5–2.7)	4.8(4.0–5.7)	10.2(8.9–11.5)	13.4(12.0–14.9)	16.0(14.4–17.6)	**0.35(<0.001)**
Total	2.2(1.7–2.6)	1.1(0.8–1.4)	2.8(2.3–3.3)	5.3(4.7–6.0)	7.0(6.2–7.7)	8.2(7.4–9.0)	**0.34(0.003)**
**North East**							
Male	0.0(0.0–0.0)	0.3(0.07–0.8)	0.0(0.0–0.0)	0.0(0.0–0.0)	0.0(0.0–0.0)	0.0(0.0–0.0)	**–0.6(0.6)**
Female	2.0(0.9–3.1)	3.2(1.8–4.7)	1.7(0.7–2.7)	4.8(3.1–6.6)	5.5(3.6–7.4)	12.1(9.3–14.9)	**0.38(0.008)**
Total	1.0(0.4–1.6)	1.8(1.0–2.5)	0.8(0.3–1.3)	2.4(1.5–3.3)	2.8(1.8–3.7)	6.1(4.7–7.5)	**0.37(0.01)**
**Desert**							
Male	0.2(0.1–0.3)	0.07(.01–0.1)	0.1(0.09–0.1)	0.04(0.008–0.1)	0.2(0.1–0.4)	0.6(0.4–0.8)	**0.35(0.5)**
Female	6.5(5.8–7.2)	3.3(2.8–3.8)	5.6(5.0–6.2)	5.2(4.6–5.8)	7.9(7.2–8.6)	18.2(17.1–19.3)	**0.27(0.001)**
Total	3.0(2.7–3.3)	1.5(1.3–1.7)	2.6(2.3–2.8)	2.4(2.1–2.7)	3.7(3.3–4.0)	8.4(7.9–8.9)	**0.27(0.03)**
**Flat**							
Male	0.01(0.005–0.06)	0.1(0.09–0.2)	0.2(0.1–0.3)	0.2(0.1–0.3)	0.2(0.1–0.4)	0.4(0.2–0.5)	**0.37(0.5)**
Female	2.7(2.3–3.1)	3.6(3.1–4.1)	4.1(3.5–4.6)	7.3(6.6–8.0)	10.7(9.9–11.6)	22.3(21.1–23.5)	**0.46(<0.001)**
Total	1.2(1.0–1.4)	1.7(1.4–1.9)	1.9(1.7–2.2)	3.4(3.1–3.7)	5.0(4.6–5.4)	10.3(9.7–10.8)	**0.46(0.001)**
**Mountainous**							
Male	0.2(0.1–0.3)	0.2(0.1–0.4)	0.3(0.1–0.4)	0.4(0.2–0.6)	0.3(0.1–0.5)	0.1(0.1–0.5)	**–0.01(0.9)**
Female	2.1(1.7–2.5)	6.0(5.3–6.7)	7.8(7.0–8.6)	8.2(7.4–9.0)	11.4(10.5–12.4)	14.6(13.5–15.6)	**0.28(0. 001)**
Total	1.1.(0.9–1.4)	3.1(2.7–3.5)	4.0(3.6–4.4)	4.3(3.8–4.7)	5.8(5.3–6.3)	7.5(7.0–8.1)	**0.28(0.02)**
**Persian Gulf**							
Male	0.0(0.0–0.0)	0.1 (0.09–0.2)	0.1 (0.09–0.2)	0.2(0.08–0.4)	0.0(0.0–0.0)	0.04(0.08–0.1)	**–2.9(1.00)**
Female	0.2(0.09–0.5)	1.3(0.8–1.8)	1.6(1.1–2.2)	2.9(2.2–3.7)	3.4(2.6–4.2)	4.5(3.6–5.5)	**0.39(0.02)**
Total	0.1 (0.09–0.2)	0.7(0.4–0.9)	0.8(0.6–1.1)	1.5(1.1–1.9)	1.7(1.3–2.1)	2.2(1.8–2.7)	**0.37(0.1)**
**Iran**							
Male	0.1(0.1–0.2)	0.1(0.1–0.2)	0.3(0.3–0.4)	0.4(0.3–0.4)	0.4(0.3–0.5)	0.5(0.4–0.6)	**0.30(0.5)**
Female	3.4(3.2–3.6)	3.9(3.6–4.1)	8.6(8.3–9.0)	11.7(11.3–12.0)	12.8(12.4–13.1)	16.7(16.3–17.1)	**0.30(<0.001)**
Total	1.8(1.7–1.9)	2.0(1.9–2.1)	4.5(4.3–4.6)	6.0(5.8–6.2)	6.5(6.3–6.7)	8.6(8.3–8.8)	**0.30(0.01)**

**Figure 1:**
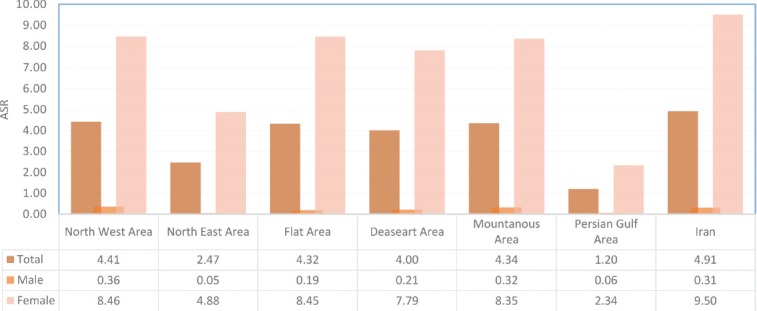
Average of age standardized incidence rate of breast cancer in 6 geographical areas of Iran during 2000–2005.

The North West region has the highest rate of breast cancer in average and the lowest average rate belongs to the region in the border of Persian Gulf (Figure 1).

### North West Area

**Figure 2:**
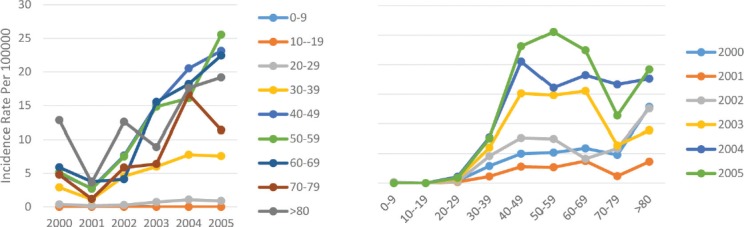
Age standardized rate of breast cancer in Center and Western border of Caspian by age groups and years.

If we calculate the mean incidence rate of breast cancer in the 6 years of study and rank different geographical areas in this regard, the western and central area of the Caspian Sea will be ranked first and its mean incidence rate will be slightly lower than that of the country (Figure 2). In this regard, this area was considered the most dangerous geographical area of Iran for the incidence of breast cancer. In this area, the age standardized incidence rate of breast cancer had risen from 2.2 per 100,000 people in 2000 to 8.2 per 100,000 people in 2005, which is a significant upward trend. But it should be noted that in 2001, in contrast to other years, the incidence rate decreased by half of the rate of the previous year and it then increased in 2002. This change trend rapidly increased since 2002 and reached its maximum in 2005. Regarding sex differentiation, it was observed that the incidence trend in both sexes had varied changes. In males, the age standardized incidence rate had changed from 7.4 cases in 2000 to 18.4 in 2005, and in female it changed from 2.9 per 100,000 in 2000 to 6.1 per 100,000 in the year 2005, with a steeper increase in male individuals (Table 1).

Of course, in all the years of study, the incidence rate in men was higher than in women, and in this regard, it was similar to the incidence trend in the whole country. In the analysis of the incidence of breast cancer in the age subgroups of this area, it was observed that in the year 2001, the incidence decreased in all subgroups and then it had a steady upward trend until 2005. Meanwhile, the incidence rate of breast cancer increased with age and reached the maximum in the age group of 40–49-years-old and decreased after the age 60–69 years. A sharp decline was then observed at the age of 70–79 years, after which the incidence rate increased (Figure 2).

### North East Area

In the eastern border of the Caspian Sea, the average 6-year incidence of breast cancer was lower than the average rate in the country and in other geographical areas, second to the Persian Gulf border. It is said that among the six geographical areas, this area was ranked the fifth in terms of the risk of breast cancer. In this area, the age standardized incidence rate increased from 1 per 100,000 in 2000 to 6.1 per 100,000 in the year 2005, and contrary to the central and western border of the Caspian Sea where the incidence rate decreased in the year 2001 and then increased, a decline was seen in this area in 2002, and thereafter we constantly saw an upward trend.

In men, except for one case in the age group 40–49-years-old and one in the age group 50–59-years-old in 2001, no new case of breast cancer was observed in the other years, and the incidence rate was zero in the year 2005, but the standard incidence rate in women increased from 2 cases per 100,000 in 2000 to 12.1 per 100,000 in 2005, which was statistically significant (Table 1). In this area, there was a sharp and sudden drop in the incidence of breast cancer in 2002, and since then, the incidence rate had an increasing trend up to the year 2005.

**Figure 3:**
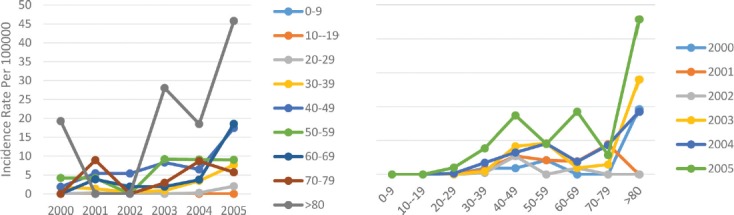
Age standardized rate of breast cancer in Eastern border of Caspian by age groups and years.

Unlike this geographical area, in the western border of the Caspian Sea, a sharp decline was seen in 2001. In the analysis of the subgroups, it was observed that in two age groups of 0–9 and 10–19 years, no breast cancer case was seen in the years of study except for one case in 2002. In this geographical area, in most of the years we observed an irregular increasing trend in the incidence of the disease among the age subgroups. The change slope was sharper after the age of 20–29 years, and the highest incidence rate occurred in the age groups 40–49 years and 60–69 years (Figure 3).

### Mountainous Area

**Figure 4:**
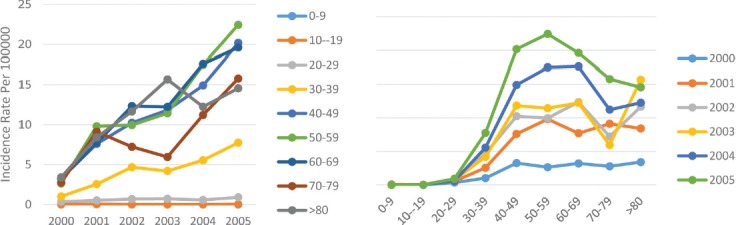
Age standardized rate of breast cancer in mountainous area by age groups and years.

After the central and western border of the Caspian Sea as the most dangerous geographical areas of Iran, the second highest incidence rate of breast cancer was seen in this area. Over the 6 years of study, we witnessed an increasing incidence of breast cancer in this geographical area. Overall, the incidence rate of breast cancer in this area changed from 1.1 per 100,000 people in 2000 to 7.5 per 100,000 in 2005, but this trend was declining in men. It means, the incidence rate first decreased from 0.2 per 100,000 men in the year 2000 to 0.4 per 100,000 in 2003, and since then, it had a declining trend again, reaching 0.1 per 100,000 in 2005, which was half the rate in 2000. In contrast, the age standardized incidence rate in women had a steady upward trend, rising from 2.1 per 100,000 in 2000 to 14.6 per 100,000 in 2005. The incidence trend analysis over the years under study in the age subgroups indicated that there was no significant change in the age groups below 20 years of age, but after the age 30–39 years, the incidence trend had a sharper slope and it became sharper in the higher ages. The incidence rate in the lower subgroups was minimal, and with age increase, we observed an increase in the incidence of breast cancer, reaching its maximum at the age of 50–59 years, and then decreasing again (Figure 4).

### Desert Area

**Figure 5:**
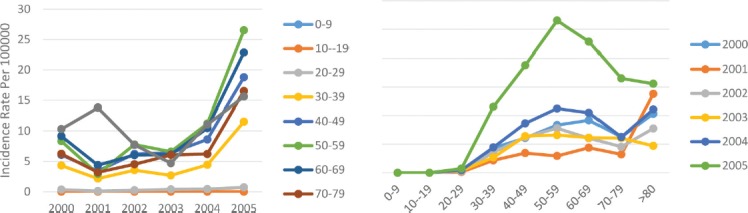
Age standardized rate of breast cancer in Desert area by age groups and years.

Comparing the 6-year averages of the incidence rate in the six geographical areas, it was showed that the desert area ranked fourth, and after the Persian Gulf border and the eastern border of the Caspian Sea, it was one of the low-risk regions of Iran in terms of the incidence of breast cancer. The overall trend of the age standardized incidence rate of breast cancer in this area was upward, like that of other areas of the country, and increased from 3.0 per 100,000 people in 2000 to 8.4 per 100,000 in 2005. The incidence rate of breast cancer in this area declined in 2001, then increased in 2002, and then decreased relatively in 2003 again but since then, it started to increase until 2005. In men, the age standardized incidence of breast cancer rose from 0.2 per 100,000 in 2000 to 0.6 per 100,000 in 2005, while this rate in women increased from 6.5 per 100,000 in 2000 to 18.2 in 100,000 in 2005 (Table 1). In the study of the incidence trend variations in the age subgroups, it was observed that in the lower subgroups, the variation trend was very slight, and with the age increase, the slope became steeper. However, in nearly all age groups, the incidence declined in the year 2001 and then increased, reaching its highest level in the year 2005. In the age groups below 30 years, breast cancer rarely occurred and after the age 30–39 years, the incidence of the disease increased, reaching its maximum rate at the age of 50–59 years, and then decreased (Figure 5).

### Flat Area

**Figure 6:**
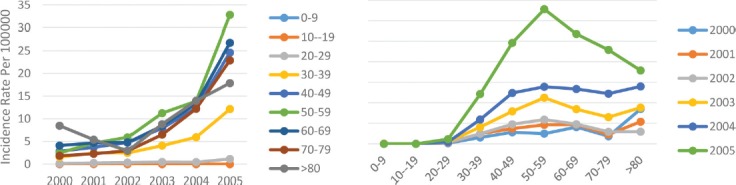
Age standardized rate of breast cancer in flat area by age groups and years.

Second to the central and western borders of the Caspian Sea and the mountainous area, this area is the third most dangerous region in terms of breast cancer in Iran. Like in other areas, we can witness an increasing incidence of breast cancer so that the age standardized incidence rate of the disease in this area rose from 1.2 per 100,000 people in 2000 to 10.3 per 100,000 in 2005. The incidence trend in this area was steadily increasing and the change slope was sharper after the year 2004. The trend of the change in the age standardized incidence was similar in both genders and they have the same incremental rate pattern. While the incidence rate in men changed from 0.01 per 100,000 in 2000 to 0.4 per 100,000 in 2005, it rose from 2.7 per 100,000 in 2000 to 22.3 per 100,000 in 2005 in women (Table 1). In this area, the incidence of breast cancer rarely occurred under the age of 20; the incidence change trend had a sharper slope with increasing age, and in most age groups the incidence rate decreased in 2002 and then increased, reaching its maximum rate in 2005. As the age rose, the incidence of breast cancer increased and reached its peak at the ages of about 50–59 years, and then increased (Figure 6).

### Persian Gulf Area

**Figure 7:**
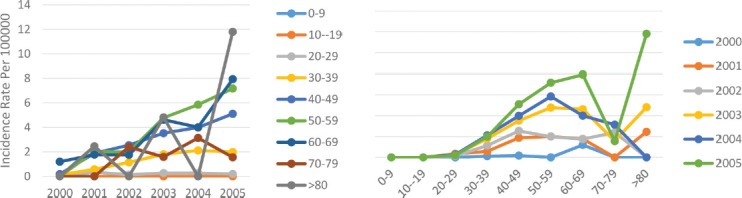
Age standardized rate of breast cancer in the border of Fars gulf area by age groups and years.

This area is the least dangerous on in Iran for the incidence of breast cancer. Compared to the central and western parts of the Caspian Sea as the most dangerous geographical area of Iran for the incidence of breast cancer, the mean 6-year incidence rate of the disease in this area was approximately 3.6 times lower. In this area, the trend of changes in the incidence over the years of study was incremental and the slope of these changes had a more constant trend compared to other geographical areas of Iran. The age standardized incidence rate in this area rose from 0.1 per 100,000 people in 2000 to 2.2 per 100,000 in 2005. In males, the age standardized incidence rate of breast cancer increased from 0.0 per 100,000 in 2000 to 0.04 per 100,000 in 2005, while in women, this rate was 0.2 per 100,000 in 2000 which reached 4.5 per 100,000 in 2005 (Table 1). Regarding age subgroups, except for the age group over 80 years in which irregular and frequent trends could be observed, there was an almost constant increasing trend in other age groups. At the age under 20, there was no case of breast cancer in any of the years and the changes in the incidence trend were variable in each age group, but in general, there was an increasing trend in all age groups. As the age increased, the incidence of breast cancer also increased, reaching its maximum rate at the age of 50–59 years, and then declined until the age of 70–79 years, and then it increased again (Figure 7).

## Discussion and Conclusion

The results of this study indicated an increase in the incidence of breast cancer in Iran and all its geographical areas. In the interpretation of the results for cancer studies in Third World countries, two fundamental issues must be taken into consideration. Firstly, if these changes are assumed to be real, in exposure to which groups of risk factors in the country was the main reason for the changes lead to an increased incidence rate, and ultimately, what proportion of the changes is justified by environmental factors and what proportion is justified by genetic factors? Secondly, a part of these changes may not be real and resulted in the improvement of the cancer registration system in the last decades in Iran. However, the clarification of this issue requires a lot of studies that carefully assess various aspects of the registration system. Here, the results are first interpreted in terms of the risk factors for breast cancer.

A recent survey of the breast cancer incidence in the world showed that the incidence has been rising rapidly in developing countries in recent decades, while in developed countries; these changes have been slighter [1]. The trend of breast cancer incidence in the United States has been declining since 1990, which is the result of two major strategies for early diagnosis of new cases of the disease and the progression of the therapies [3]. In Europe, the fatality rate for this cancer has declined, and the main reason for this has been considered the therapeutic use of anti-estrogens and chemotherapy [27, 28]. Besides, the incidence rate of the disease also declined as a result of the reduction in the use of hormone therapy after the publication of a related study in 2002 [29]. On the contrary, the incidence rate of breast cancer in Asian countries is rising. Several justifications have been raised for the increased incidence of the disease in Asia, including low age of menarche, delayed menopause, reduced fertility, increased first-delivery age, weight gain, and dietary changes [30]. They can all be considered among the known risk factors for this cancer, and the wave of exposure to them has reached the Asian countries after the West World, with decades of delay. Comparing the results of this study with the situation in other Asian countries indicated that the incidence rate in Iranian women increased to over 16 per 100,000 people in 2007, while in Pakistan, Singapore and the Philippines it was respectively 50.1, 48.7 and 46.4 per 100,000, indicating a far higher incidence rate of the disease in these countries than in Iran. In contrast, there are other countries where the incidence of breast cancer is lower than in our country, including Mongolia and Laos with incidence rates of 6.6 and 10.9 per 100,000 people, respectively [31]. A comparison with previous studies showed that in Iran, breast cancer has had an increasing trend. A study on cancers in Tehran in 1970 indicated that breast cancer accounted for only 5% of all cancers and was the fifth most common cancer in Iran [16], all of which indicate the consistency of the results of the present study with the status of the incidence of breast cancer in most Asian countries.

In Iran, demographic policies have led to a decrease in the birth rate in women, which is one of the risk factors for the growth of breast cancer. In addition, the socioeconomic conditions in recent decades have caused the average age of marriage to rise in Iran, and it can be said that the combination of these factors, which intensify each other’s effects, has been one of the causes of an increase in the incidence of breast cancer in the country. Along with the abovementioned factors, it is even possible to mention the reduced rate of breastfeeding in women due to caring for their physical beauty and the use of complementary foods for their babies [32], it is suggested that breastfeeding could be one of the most promising factors in the prevention of breast cancer in women [1, 3], and domestic research have also confirmed it [7]. After the Islamic Revolution of Iran, an increasing urbanization was seen, and two relatively different types of life could be imagined for the women in rural and urban areas. In the urban women, the risk factors for breast cancer such as nutrition, marriage age, breastfeeding rates and the use of estrogen pills were far higher than in the rural women.

One of the important variables that should be considered in the study of cancers, and especially breast cancer, is the incidence age of this cancer. In general, in Asian countries, breast cancer occurs at earlier ages compared to the Western world, and the age group 40–49 years is the most common group, and the average age of 50 is the peak age of breast cancer [33, 34]. Comparing the results of the present study for different geographical areas in Iran suggests that, except for the northern regions of the country, at an approximate age of 40–49 years, the highest rates of breast cancer might be seen in all other regions of the country and the peak rate of the disease is somewhat higher than in other East Asian countries, which could be due to the difference in the age pyramid of these countries. However, in Iran, after an approximate age of 55, the incidence of breast cancer declines again, the most important reason for which could be the cultural and social problems of the people for early referrals and correct diagnosis, compared to the Western countries [35]. In this regard, a study on the survival rate of Iranian breast cancer patients showed that about 60% of the patients were diagnosed in stages 3 and 4 of the disease progression, and the early diagnosis of this cancer and the use of standard therapies would help increase the survival rate of the patients [36]. It should also be noted that reduced use of estrogen therapy at a higher age [37] could somewhat decrease the incidence of the disease after the age of 50. However, the increased mean age and the aging phenomenon could also be a factor in the overall increase of the incidence of skin cancer and other cancers in Iran because, according to the results of the study on the indicators of heath feature in Iran, the life expectancy in this country has increased [38], and since the incidence of skin cancer was also the highest in the 1980s, the rise in the incidence of this cancer in Iran could be somewhat justified.

The changes in lifestyle and nutrition are among the factors that increase the overall incidence of cancers, including breast cancer [39]. Previous studies in Iran showed increasing trend for stomach cancer [40, 41], skin cancer [42, 43], colorectal cancer [44, 45], lung cancer [46], brain [47]. In-line with globalization and changing the nutritional pattern of eastern countries from high-vegetable and low-calorie foods to high-fat and high-calories, Iran is also witnessing such changes in the nutritional pattern of its people, and studies showed the increasing trend of the risk factors for cancers in this country [48]. Regarding breast cancer, it could be seen that the incidence of the disease has increased in Asian migrants to America [49].

One of the most important factors in increasing the incidence of breast cancer in the world and in Iran is the implementation of a screening program for quick diagnosis of new cases at the earliest possible time. The easiest way to screen for this cancer is mammography, which detects about 80%–90% of breast cancer patients without clinical symptoms [3]. The program is also ongoing in Iran, and evidence suggests that despite achievements in this country, there are also some problems with the screening program, and it is also evident that regular participations in routine breast cancer screening programs are not done [19]. Women delay their next visits [20], and although health staff have proper knowledge and attitude towards this disease, they are less likely to use a screening program [21]. In the screening program in Iran, the highest participation rate is that of the women aged 35–44 years and from a middle socioeconomic class, and the lowest participation rate is seen among the women over the age of 60 and from a low socioeconomic class. Also, the rate of screening in the self-assessment method is similar to that of the assessment by health personnel [23]. Ordinary women have very low knowledge of the symptoms of breast cancer and its screening program, and there is still a strong need for educating them [25].

It should be noted that the address of a large number of breast cancer cases registered in the cancer registry system of the country is unknown, and therefore, they have not been included in any of the geographical areas of the country, but they were included in the calculation of the overall incidence rate of breast cancer in Iran. Hence, it can be concluded that the calculated incidence rate for Iran is not the result of the mean incidence rates in all geographical areas, but somewhat higher. This point should be considered in the analysis of the results. The global burden of disease cancer collaboration (GBD-CC) and GLOBOCAN projects also have the same problem with the quality of the data in developing country which in the case of Iran lead to under estimating [50].

The most important issue in interpreting the results of this study and justifying the increasing trend of breast cancer in Iran is the improvement of the cancer registry system in the country. The study of the incidence trend of cancers in Italy showed that the increase in the incidence of this cancer was largely related to the development of diagnostic techniques and the development of the cancer registration system [51]. In Iran, both the registry and the diagnosis systems have problems that must be addressed [15]. The National Cancer Registry System of Iran was established under the supervision of Tehran University of Medical Sciences in 1984, and since 1999 the data of this registry system have been published [52].

The cancer registry system in Iran has had an increasing trend in recent years [52], and according to the Ministry of Health, the cancer registry rate in the country has increased from 18% in 1999 to 80% in 2005 [53], which might be due to a high increase in the incidence of breast cancer and other cancers in Iran. Therefore, it is very important to consider the problems and the evolutionary process of cancer registry system when interpreting the results of all cancer studies in Iran. But the rate of this increase and the geographical areas where the increase is higher are the issues that should be considered in future research, and it is natural that such a trend exists more or less in most countries.

## Conflict of Interest

The authors confirm that there are no conflicts of interest.
